# Plasma antioxidant capacity in critical polytraumatized patients?: methods, severity, and anatomic location

**DOI:** 10.1186/cc13917

**Published:** 2014-06-12

**Authors:** Luis Serviá, Javier Trujillano, José Carlos Enrique Serrano, Reinald Pamplona, Mariona Badia, Mariona Jové, Margarida Justes, Joana Domingo, Manuel Portero-Otin

**Affiliations:** 1 Department of Critical Care Unit, 6th floor, University Hospital Arnau de Vilanova, Rovira Roure 80, 25198 Lleida, Spain; 2 NUTREN-Nutrigenomics, Biomedical Research Institute (IRB) of Lleida-UdL, Biomedicine I Building, Office 3.13, Rovira Roure 80, 25198 Lleida, Spain

## Findings

Oxidative stress (OS) has been invoked as a relevant factor in the evolution and outcome of critical care patients. Indeed, antioxidant therapies have been used in critical care patients [1] but with controversial results [2]. This may be explained by assuming OS as a homeostatically regulated parameter and both its excess and its deficit influencing severity progression. Nonetheless, antioxidant agents could mask an OS signaling role, blocking otherwise physiological responses aimed at recovery of homeostasis [3]. We have evaluated plasma total antioxidant capacity (TAC) in traumatized patients in an ICU, and we determined its potential relationship with severity and trauma location. In a prospective observational study of ICU polytraumatized patients (n = 73, mean Acute Physiology and Chronic Health Evaluation II (APACHE II) score of 11 ± 6) of the Hospital Arnau de Vilanova (Lleida, Spain), we measured (in the first 48 hours) plasma TAC by two different methods: the ferric reducing activity/antioxidant power (FRAP) and the capacity for neutralization of the free radical 2,2′-azino-bis (3-ethylbenzothiazoline)-6-sulphonic acid (ABTS) as previously described [4]. For control subjects, we used age- and gender-matched volunteers (n = 102). We also evaluated the contribution of antioxidant molecules (uric acid, bilirubin, and albumin) to these values. The protocol was approved by the institutional ethics committee of the Arnau de Vilanova Hospital and followed Declaration of Helsinki guidelines for studies with human individuals. All participants (or their legal representatives) gave their consent for the study.

## Results

**Figure 1:**
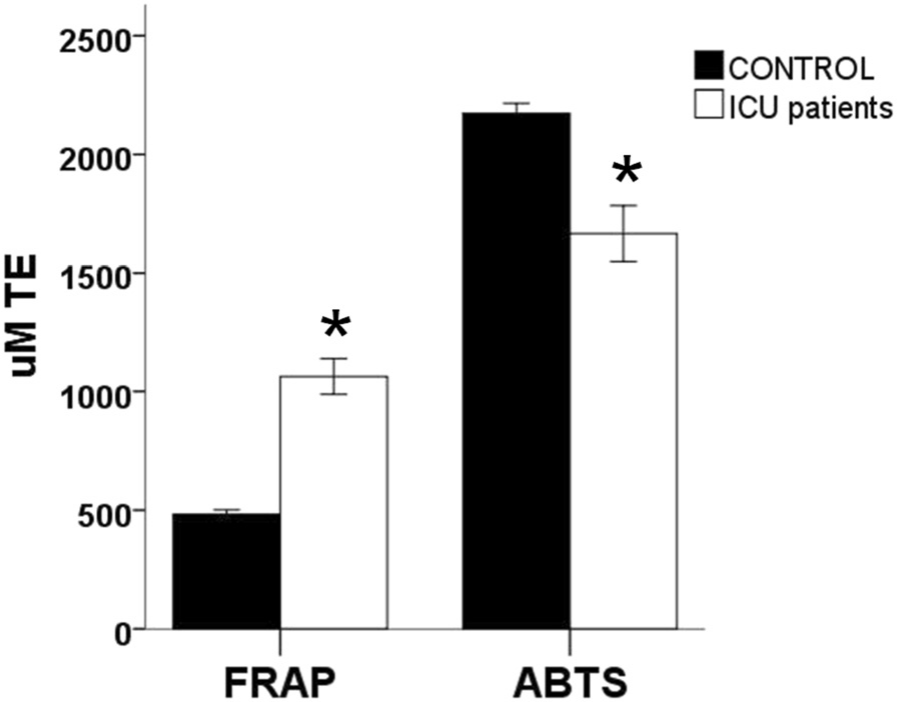
**FRAP and ABTS values in plasma of ICU polytrauma patients (n = 73) are significantly different from those of an age- and gender-matched population (CONTROL) (n = 102).** Asterisk indicates statistical differences according to Mann-Whitney test (*P* <0.05). FRAP and ABTS in plasma were measured as described [4] by using, respectively, 2,4,6-tri(2-piridil)-s-triazine, FeCl_3_, and acetate buffer (300 mM, pH 3.6) and the ABTS radical cation (ABTS + ·) as reagents and by using a Beckman DU-640 spectrophotometer (Beckman Instruments Inc., Fullerton, CA, USA). Both capacities were referenced to standards containing known concentrations of 6-hydroxy-2,5,7,8-tetramethylchroman-2-carboxylic acid (Trolox) and expressed as micromolar trolox equivalents (TE). ABTS, 2,2′-azino-bis (3-ethylbenzothiazoline)-6-sulphonic acid; APACHE II, Acute Physiology and Chronic Health Evaluation II; FRAP, ferric reducing/antioxidant power; OS, oxidative stress; TAC, total antioxidant capacity.

As shown in Figure 1, polytraumatized patients show differences in TAC with reference to control subjects, but these differences are dependent on the technique used. Thus, ICU polytraumatized patients show higher FRAP values but lower ABTS capacity. Notably, APACHE II score influenced FRAP values (Table 1). Indeed, we found that FRAP values were inversely correlated with APACHE II score (*r* = -0.266, *P* <0.01) suggesting that, in trauma patients, increased antioxidant response, as measured by FRAP assay, could be a pathophysiological response to stress. Albumin and uric acid concentrations reproduced the FRAP trend with severity. Reinforcing the importance of the technique and the specificities across different antioxidant assessment methods, data for the relationship of APACHE II score with ABTS do not show a significant trend (*r* = 0.040, *P* = 0.568). These results also contrast with those obtained in other ICU patients, such as those with sepsis [5].

**Table 1:** Study population characteristics of ICU polytraumatized patients

**Study population characteristics**	**APACHE II score**			***P *** **value**
	**≤7 (n = 28)**	**8-14 (n = 23)**	**≥15 (n = 22)**	
Age, years^a^	42.4 ± 14	49.9 ± 20	49.2 ± 19	0.186
Male gender	78.6	82.6	81.8	0.761
AIS (≥3)				
Head	17.9	43.5	54.5	0.045
Chest	57.1	65.2	40.9	0.297
Abdomen	10.7	8.7	18.2	0.503
FRAP, μM TE^b^	1,093 (1,013-1,286)	1,072 (856-1,222)	825 (649-1,250)	0.044
ABTS, μM TE^b^	1,763 (1,555-2,035)	1,765 (1,054-2,008)	1,825 (1,405-1,982)	0.852
Uric acid, mg/dL^b^	3.8 (2.0-5.9)	2.5 (1.5-4.6)	2.1 (1.2-4.6)	0.056
Bilirrubin, mg/dL^b^	0.6 (0.4-0.9)	0.8 (0.5-1.4)	0.6 (0.5-1.2)	0.104
Albumin, mg/dL^b^	3.6 (3.1-3.7)	3.3 (2.7-3.6)	3.0 (2.6-3.4)	0.016
ICU mortality	0.0	0.0	13.6	0.027

Severity was staged according to Acute Physiology and Chronic Health Evaluation II (APACHE II) score. Values are presented as a percentage unless indicated otherwise. ^a^Mean ± standard deviation; ^b^median (interquartile range). *P* according to χ^2^ or Kruskal-Wallis test between different APACHE II groups. ABTS, 2,2′-azino-bis (3-ethylbenzothiazoline)-6-sulphonic acid; AIS, abbreviated injury scale; *FRAP*, ferric reducing/antioxidant power; *ICU*, intensive care unit; *TE*, micromolar trolox equivalents.

In the multiple linear regression, FRAP values in trauma ICU patients are independently influenced by age (β = 0.271, *P* <0.021), APACHE II score (β = -0.356, *P* <0.002), and head trauma (β = -0.219, *P* <0.045). These results accentuate the influence of trauma location and severity in TAC changes.

Our results not only stress the importance of the method used for TAC measurement but also show that age, status severity, and anatomical location of trauma influence TAC response in ICU patients, reinforcing the need for an adequate tailoring of treatments aimed at their recovery, such as antioxidant therapies.

## Abbreviations

ABTS: 2,2′-azino-bis (3-ethylbenzothiazoline)-6-sulphonic acid; APACHE II: Acute physiology and chronic health evaluation II; FRAP: Ferric reducing/antioxidant power; OS: Oxidative stress; TAC: Total antioxidant capacity.

## Competing interests

The authors declare that they have no competing interests.
